# Factors influencing intention to use AI in business decision making among professional employees in Malaysia’s financial industry

**DOI:** 10.3389/frai.2026.1779573

**Published:** 2026-03-24

**Authors:** Gehad Mohammed Ahmed Naji, Mohammed Alzoraiki, Nagendra Suba Rao, Yulita Hanum P Iskandar

**Affiliations:** 1 Graduate School of Business, Universiti Sains Malaysia, George Town, Malaysia; 2 Administrative Science Department, College of Administrative and Financial Science, Gulf University, Sanad, Bahrain

**Keywords:** artificial intelligence, industrial growth and digital transformation, intention to use, technology acceptance model, value-based adoption model

## Abstract

**Purpose:**

This research investigates the factors influencing professional employees’ intentions to use AI in business decision-making within Malaysia’s financial industry by integrating the Technology Acceptance Model (TAM) with the Value-Based Adoption Model (VAM). More specifically, the study examines how perceived usefulness, perceived ease of use, perceived enjoyment, perceived risk, perceived value, and attitude jointly influence behavioral intention to use artificial intelligence.

**Research design and methodology:**

A quantitative approach used structured online questionnaires distributed to 210 professional employees across various Malaysian financial institutions (digital financial services, banking, insurance, investment management, and provident funds). Data were analyzed using Partial Least Squares Structural Equation Modeling (PLS-SEM) via SmartPLS 4 to assess the measurement model and test the eleven hypothesized relationships.

**Findings:**

The integrated TAM–VAM framework explained 74% of the variance in AI use intention, with seven of eleven hypotheses supported. Attitude was the strongest direct predictor of intention (*β* = 0.773, *p* < 0.001). Perceived value had a significant positive effect on attitude (*β* = 0.675, *p* < 0.001) but did not directly influence intention. Perceived usefulness, perceived ease of use, and perceived enjoyment each positively affected perceived value. Perceived risk negatively influenced attitude. The results highlight perceived value and attitude as key mediators in the AI adoption process. Given the cross-sectional design, findings should be interpreted as predictive associations rather than causal effects.

**Research limitations:**

This study used convenience and snowball sampling within Malaysia’s financial sector, which may introduce selection bias and limit generalizability. The sample overrepresented FinTech employees (50.5%), who may hold more favorable views toward AI compared to employees in traditional banking or insurance. Moreover, the cross-sectional design captured perceptions at a single point in time and measured behavioral intention rather than actual usage behavior, further limiting external validity.

**Practical/theoretical implications:**

Theoretically, this research extends technology acceptance theory by validating the integrated TAM-VAM framework in emerging markets, showing that value-based mechanisms mediate utilitarian perceptions’ influence on attitudes. Findings offer valuable insights for financial institutions’ AI implementation, vendors’ user-centered solutions, and policymakers’ supportive regulatory frameworks for responsible AI use in Malaysia’s financial sector.

## Introduction

The global financial services industry is experiencing unprecedented transformation driven by artificial intelligence technologies, with applications spanning algorithmic trading, credit risk assessment, fraud detection, and automated customer service delivery (Patil, 2025). In Malaysia, the financial sector has demonstrated increasing commitment to digital transformation, supported by Bank Negara Malaysia’s progressive regulatory framework and substantial institutional investments in technological infrastructure (Mohd Rashid et al., 2023). Financial institutions across banking, insurance, investment management, and emerging FinTech segments have deployed sophisticated artificial intelligence systems intended to enhance operational efficiency, improve decision accuracy, and maintain competitive positioning in an increasingly technology-driven marketplace. Despite these organizational investments and technological capabilities, realizing the anticipated benefits of artificial intelligence implementation depends critically on professional employees’ willingness to integrate these tools into their daily work activities and business decision-making processes.

A significant gap persists between artificial intelligence deployment and actual adoption intention among professional employees in Malaysia’s financial industry. Recent evidence indicates that fewer than 30 % of implemented artificial intelligence systems achieve widespread employee acceptance within their first operational year, resulting in substantial underutilization of organizational technology investments (Dwivedi et al., 2021a,b; Klein et al., 2023). This adoption intention gap is particularly consequential in business decision-making contexts, where professional employees must voluntarily choose to rely on artificial intelligence recommendations when making judgments with significant financial implications. Unlike routine task automation that can be mandated organizationally, effective integration of artificial intelligence into professional decision-making requires genuine behavioral intention reflecting confidence, trust, and perceived value. When employees bypass available artificial intelligence tools in favor of traditional analytical approaches, organizations fail to capture returns on technology investments while simultaneously missing opportunities for enhanced decision quality, processing speed, and competitive advantage that motivated the initial artificial intelligence adoption (Liao et al., 2022).

The factors influencing this adoption intention among professional employees in Malaysia’s financial sector remain inadequately understood. While extensive technology acceptance research has been conducted in Western developed economies, limited empirical investigation has examined emerging market contexts where regulatory environments, organizational cultures, technological literacy levels, and risk perceptions may differ substantially (Mohd Rashid et al., 2023; Kok and Supaprawat, 2023). Professional employees in Malaysia’s financial industry face distinctive considerations including concerns about data privacy within evolving regulatory frameworks, uncertainties about algorithmic transparency in high-stakes decision environments, and questions about whether artificial intelligence genuinely enhances rather than complicates established professional judgment processes. Moreover, existing theoretical frameworks may inadequately capture the complexity of professional artificial intelligence adoption. The Technology Acceptance Model emphasizes utilitarian perceptions of usefulness and ease of use, yet professional adoption likely involves broader value assessments weighing functional benefits against costs including cognitive effort, perceived risks, and potential professional implications (Davis, 1989). The Value-Based Adoption Model introduces comprehensive value considerations but has been predominantly applied to consumer technology adoption rather than professional organizational settings where motivations and constraints differ fundamentally (Kim et al., 2007).

Even though organizations are investing more in AI, there is still little empirical data on how Malaysian financial professionals decide whether or not to use AI in business decisions, especially in emerging market contexts where risk sensitivity, professional accountability, and governance expectations may differ from those in Western settings. Furthermore, value-based trade-offs (benefits vs. sacrifices) that experts take into account when depending on AI suggestions in high-stakes financial decisions may not be adequately captured by current TAM-based studies. By combining TAM and VAM to explain AI usage intention through perceived value and attitude, Our work addresses these gaps. The research pursues three specific objectives. First, to identify and examine the relationships between perceived factors (usefulness, ease of use, enjoyment, risk) and adoption intention through value and attitude formation. Secondly, to analyze the mediating roles of perceived value and attitude in translating antecedent perceptions into behavioral intentions. Third, to provide empirically grounded recommendations for Malaysian financial institutions, technology vendors, and policymakers seeking to enhance artificial intelligence adoption effectiveness.

This research offers significant theoretical contributions by extending established technology acceptance frameworks to contemporary artificial intelligence adoption in emerging market professional contexts.

The integration of Technology Acceptance Model and Value-Based Adoption Model provides a comprehensive theoretical lens that captures both utilitarian and hedonic dimensions of professional technology acceptance, while incorporating value-based trade-offs increasingly relevant for complex artificial intelligence systems. By empirically validating this integrated framework within Malaysia’s financial sector, the study demonstrates cross-cultural applicability of acceptance theories while revealing context-specific patterns that advance understanding of professional artificial intelligence adoption mechanisms. The mediating role analysis of perceived value and attitude illuminates psychological pathways through which functional beliefs translate into behavioral intentions, contributing theoretical depth to adoption process understanding (Dwivedi et al., 2021a,b; Klein et al., 2023).

Furthermore, this research provides actionable insights for financial institutions implementing artificial intelligence decision support systems. Understanding the specific factors to influencing intention enables organizations to design targeted change management strategies, optimize user training programs, and configure systems that align with professional needs and preferences. For technology vendors developing artificial intelligence solutions for financial services, the findings inform user-centered design principles that enhance acceptance likelihood. For policymakers and regulatory bodies such as Bank Negara Malaysia, this study offers evidence based guidance for creating supportive frameworks that balance innovation encouragement with appropriate risk governance, thereby facilitating responsible artificial intelligence integration across Malaysia’s financial industry (Mohd Rashid et al., 2023). Enhanced understanding of adoption drivers can accelerate digital transformation effectiveness, improve return on technology investments, and strengthen Malaysia’s competitive positioning in the evolving global financial services landscape.

A good understanding of the key determinants influencing adoption, such as how the perceived ease of use or perceived enjoyment of AI tools influences employees’ attitudes and intentions, will provide managers with actionable insights that can be directly applied in organizational strategies (Qadri et al., 2024). For example, if perceived risk emerges as a significant inhibitor, organizations may implement stronger data security protocols and user training programs. Conversely, if perceived ease of use is shown to influence perceived value, then developing intuitive and user-friendly systems should be prioritized. Ultimately, the study’s outcomes will guide the formulation of strategies that promote widespread adoption of AI, leading to improved decision making, higher efficiency, and a competitive edge in the financial market.

## Literature review

### Introduction

Artificial intelligence (AI) is reshaping financial decision making by enabling data-driven, timely insights. Yet organizations frequently struggle to move from pilot AI projects to widespread adoption (Dwivedi et al., 2021a). In the rapidly evolving landscape of technology, the focus will be on models that are particularly relevant to understanding the adoption of artificial intelligence (AI) in business decision- making within the financial industry. The chapter will primarily explore two prominent theoretical frameworks: the Technology Acceptance Model (TAM) and the Value- Based Adoption Model (VAM). The integration of these two models will be justified as a means of providing a more holistic understanding of the factors influencing the intention to use AI. Following this introduction, the chapter will delve into a detailed discussion of the underlying theories, the rationale for integrating TAM and VAM, a review of the dependent variable (intention to use AI), and a systematic analysis of the independent variables identified as potentially influencing this intention. The chapter will then present the research framework that guides this research, culminating in the development of specific research hypotheses that will be tested in the subsequent stages of the study. This chapter provides a thorough examination of the factors that influence professional employees in the Malaysian financial sector to actually use AI for business decisions. Previous research on the adoption of AI in finance frequently relies on TAM constructs (usefulness, simplicity of use), often relies on TAM constructs but fails to adequately describe the value trade-off process professionals carry out when adoption entails uncertainty, accountability, and compliance (Adamek and Solarz, 2025; Naji et al., 2025a). Although VAM offers a value-based evaluation lens, consumer contexts have seen its application more often than professional decision-support systems. Therefore, in Malaysia’s financial sector, it is still unclear whether TAM beliefs directly influence professionals’ attitudes or largely through perceived value, and whether perceived value predominantly or directly predicts intention through attitude.

### Technology acceptance model (TAM)

In information systems research, the Technology Acceptance Model (TAM), first put forth by Davis in 1989, continues to be a highly significant framework for understanding user acceptance and technology utilization. TAM, which was developed from Fishbein and Ajzen's (1975) Theory of Reasoned Action (TRA), streamlines TRA by concentrating on the factors that influence technology adoption in organizational settings. It asserts that a person’s attitudes towards a technology largely influence their intention to use it. Perceived Usefulness (PU) and Perceived Ease of Use (PEOU), two fundamental cognitive assumptions initially identified by Davis (1989), in turn influence these attitudes. These concepts are also widely discussed in subsequent reviews of adoption models (Taherdoost, 2018).

Perceived Usefulness (PU) refers to “the degree to which a person believes that using a particular system would enhance his or her job performance” (Davis, 1989). It captures the user’s assessment of the technology’s instrumental value in achieving work-related goals, such as improving efficiency, effectiveness, or overall productivity, a point reinforced in studies applying TAM to contexts like M- learning (Al-Emran et al., 2018). Perceived Ease of Use (PEOU) is defined as “the degree to which a person believes that using a particular system would be free of effort” (Davis, 1989). This construct relates to the user’s perception of the technology’s complexity; systems perceived as less complex and easier to operate are more likely to be adopted, as noted by Taherdoost (2018).

TAM proposes specific relationships among these constructs. Davis (1989) theorized that PEOU positively influences PU, suggesting that technologies requiring less effort to use are also more likely to be perceived as useful. Both PU and PEOU are considered significant antecedents of Attitude towards Use (ATT), representing the user’s overall positive or negative evaluation of using the technology.

Attitude, subsequently, directly influences Behavioral Intention to Use (BI). This intention is considered the most proximal predictor of actual system use according to Davis (1989) and confirmed in later reviews (Lai, 2017).

TAM’s enduring appeal stems from its parsimony and strong empirical support across diverse technological domains and user groups. Reviews such as Lai (2017) highlight this predictive power. Its focus on user perceptions provides valuable insights for predicting and managing technology implementation. However, TAM is not without limitations. Critics note its primary focus on utilitarian aspects may neglect hedonic motivations like enjoyment (der Heijden, 2004). Furthermore, it has a tendency to overlook crucial social influences and contextual factors that shape adoption behaviors, a point raised by Taherdoost (2018) and also discussed in relation to the evolution of technology adoption research (Dwivedi et al., 2021a). For complex technologies like AI, especially in high-risk environments such as finance, these limitations suggest the need for a more comprehensive model incorporating additional influencing factors.

### Value-based adoption model (VAM)

To address limitations of TAM, particularly its emphasis on utilitarian aspects, the VAM model was introduced, Kim et al. (2007) introduced the Value-Based Adoption Model (VAM). Drawing from consumer behavior theories, VAM posits that technology adoption is driven by a value-maximization perspective, where users weigh the perceived benefits against the perceived sacrifices associated with using the technology (Kim et al., 2007).

The core construct in VAM is perceived value (PV). This is defined as the consumer’s overall assessment of the utility of a product (or technology) based on perceptions of what is received (benefits) and what is given (Kim et al., 2007). This definition is consistently applied in studies integrating VAM, such as the work by Liao et al. (2022). Benefits encompass both utilitarian outcomes (e.g., usefulness, efficiency gains) and hedonic outcomes (e.g., enjoyment, intrinsic satisfaction). Sacrifices include monetary costs (though often less relevant in mandatory work contexts) and non-monetary costs such as the effort required to learn and use the technology (related to PEOU), technical complexity, and perceived risks (Kim et al., 2007). VAM suggests that users are more likely to adopt technologies they perceive as offering superior value. This value perception often mediates the influence of specific benefits and sacrifices on adoption intention, as demonstrated by Kim et al. (2007) and supported in later research (Hsiao et al., 2016).

The strength of VAM is its more comprehensive understanding of adoption drivers, which incorporates perceived risks and sacrifices together with utilitarian and hedonistic advantages (Liao et al., 2022). This makes it especially useful for researching the impact of technologies like artificial intelligence (AI), which offer sophisticated features but may also raise hazards or take a lot of work to learn. VAM offers a deeper comprehension of the trade-offs people make when determining whether to adopt a new technology by explicitly including perceived value as a key mediator (Kang et al., 2023). The depth provided by VAM is useful for comprehending the adoption of advanced technologies, even though it may be more difficult to operationalize than TAM due to the inclusion of numerous value dimensions (Kim et al., 2007).

### Justification of integrated TAM–VAM

This study proposes an integrated framework combining TAM and VAM to investigate factors influencing AI use intention among Malaysian financial industry employees. This integration is justified by the models’ complementary strengths, offering a more holistic understanding than either alone, as shown in previous studies (Liao et al., 2022; Wu and Chen, 2017).

TAM, a well-validated model, posits Perceived Usefulness (PU) and Perceived Ease of Use (PEOU) as core drivers of attitude and intention, confirmed in many reviews (Davis, 1989; Lai, 2017). While effective for initial reactions to functionality and usability, TAM’s focus on utilitarian aspects limits its scope for advanced technologies like AI, where intrinsic motivations and risk perceptions are also highly influential (Dwivedi et al., 2021a; der Heijden, 2004).

VAM complements TAM by introducing perceived value (PV) as a central construct. It explicitly incorporates the trade-off between perceived benefits (including both usefulness and enjoyment) and perceived sacrifices (such as perceived risk and effort) (Kim et al., 2007). This value-based perspective is particularly relevant for AI adoption in the financial sector. The inclusion of constructs like Perceived Enjoyment captures intrinsic motivation, while Perceived Risk addresses user concerns, providing a more nuanced view of the adoption decision (Hsiao et al., 2016; Liao et al., 2022).

By integrating TAM and VAM, this study leverages TAM’s strengths in explaining the role of core usability and usefulness perceptions. It simultaneously incorporates VAM’s broader value- oriented perspective, including hedonic aspects and risks (Liao et al., 2022). This integrated framework allows for examining how functional perceptions (PU, PEOU) and evaluative factors (Perceived Enjoyment, Perceived Risk) collectively influence perceived value and attitudes, which ultimately drive the Intention to Use AI. Such a comprehensive approach is crucial for understanding technology adoption in the high-stakes, rapidly evolving context of AI in Malaysia’s financial industry.

### Review of dependent variable: intention to use AI in business decision making

The dependent variable in this study is the Intention to Use AI in Business Decision Making. Behavioral intention is defined as an individual’s subjective probability that they will perform a specific behavior (Fishbein and Ajzen, 1975). It is considered the most direct predictor of actual technology use within models like TAM (Davis, 1989) and VAM (Kim et al., 2007). In the context of this research, it refers to the willingness and readiness of professional employees in Malaysia’s financial industry to adopt and utilize AI tools to support, enhance, or automate their decision-making processes. The financial services industry globally is undergoing significant transformation driven by AI. Applications range from algorithmic trading and credit scoring to fraud detection and personalized customer service, as reviewed and discussed in terms of advancements by Patil (2025). In Malaysia, the financial sector, guided by initiatives from Bank Negara Malaysia, has shown increasing adoption of digital technologies. This includes FinTech and AI-driven solutions aimed at improving efficiency and competitiveness (Mohsin et al., 2022; Mohd Rashid et al., 2023). Despite the recognized potential and ongoing investments, the successful integration of AI hinges on user acceptance and adoption intention. Klein et al. (2023) emphasize the importance of user perceptions in this process. Factors influencing this intention are multifaceted. They include perceptions of AI’s benefits (e.g., improved accuracy, efficiency) and drawbacks (e.g., job security concerns, lack of transparency), as discussed in broad reviews of AI challenges and opportunities (Dwivedi et al., 2021a,b). Trust in AI and perceived risks, such as data privacy and algorithmic bias, are critical (Klein et al., 2023). Identifying specific factors influencing intention among Malaysian financial professionals is essential to maximize AI investment returns and effectively leverage these technologies for better business decisions.

### Review of independent variables

#### Perceived usefulness (PU)

Perceived Usefulness (PU), defined by Davis (1989) as the belief that using a technology will enhance job performance, is arguably the most consistent predictor of usage intention across various TAM studies. Lai (2017) provides a review supporting this consistency. It reflects the user’s assessment of the technology’s extrinsic value in achieving work objectives. When users believe a technology will help them perform tasks better, faster, or more effectively, they develop more positive attitudes and stronger intentions to use it (Al-Emran et al., 2018). In the context of AI adoption, PU remains a critical factor. Studies examining AI-powered systems, such as AI assistants or AI powered or assisted trading systems, consistently find that perceptions of usefulness significantly drive adoption intentions. For financial professionals, the perceived ability of AI to improve the quality of analyses, enhance risk assessment accuracy, automate routine tasks, or provide timely insights for decision-making would constitute its usefulness (Patil, 2025). Malaysian research on related technologies, such as digital banking, frequently shows that Perceived Usefulness (PU) positively impacts intentions (Mohsin et al., 2022). Consequently, financial industry employees in Malaysia who view AI as enhancing job performance are expected to have a higher intention to use it.

#### Perceived ease of use (PEOU)

Perceived Ease of Use (PEOU) refers to the belief that using a technology will be free from physical and mental effort (Davis, 1989). TAM posits that PEOU influences intention both directly and indirectly through its positive effect on PU (Davis, 1989). This relationship is also highlighted in reviews like Taherdoost (2018). The rationale is that users are more likely to explore and utilize the features of an easy-to-use system, leading them to better appreciate its usefulness.

However, the direct impact of PEOU on intention can be context-dependent. While crucial for initial adoption, especially for less experienced users, its direct influence may diminish as users gain experience or when the perceived usefulness is extremely high (Lai, 2017). In some studies involving complex systems or professional users, PEOU’s direct effect on intention has been found to be non- significant, although its indirect effect via PU often remains. For instance, preliminary findings in Malaysia’s digital banking context suggest PEOU may not directly drive intention (Mohd Rashid et al., 2023), implying that financial professionals might prioritize performance gains (PU) over initial usability challenges. PEOU remains crucial for minimizing user frustration and cognitive load, thus retaining its role as a key antecedent potentially influencing both attitude and perceived value in the proposed framework.

#### Perceived enjoyment (PE)

Perceived Enjoyment (PE) refers to the extent to which the activity of using a technology is perceived to be enjoyable in its own right, apart from any performance consequences. This concept was notably integrated into technology acceptance research by der Heijden (2004). It captures the intrinsic motivation associated with technology use and is a key component reflecting hedonic benefits within the VAM framework (Kim et al., 2007). Numerous studies have demonstrated that PE positively influences user attitudes, perceived value, and intentions to use various technologies. Examples include research on general technology adoption integrating TAM and VAM (Liao et al., 2022), and social commerce contexts (Qadri et al., 2024). While financial decision-making is primarily a utilitarian task, the role of intrinsic motivation should not be underestimated, even in professional settings. If interacting with AI tools—perhaps through intuitive interfaces, engaging data visualizations, or the satisfaction of mastering a sophisticated system—is perceived as enjoyable or interesting, it can foster more positive attitudes. It can also increase the perceived value beyond mere functionality (Qadri et al., 2024). This intrinsic appeal can encourage exploration, learning, and ultimately, sustained use of AI tools.

Therefore, PE is included as a potential driver of both attitude towards using AI and the overall perceived value derived from it.

#### Perceived risk (PR)

Perceived Risk (PR) refers to a user’s subjective belief about the potential for uncertain, negative outcomes associated with adopting or using a technology. This concept was explored in early e- commerce studies by Featherman and Pavlou (2003) and applied to contexts like internet banking by Kesharwani and Singh Bisht (2012). PR acts as a major inhibitor to technology adoption, negatively influencing attitudes, perceived value, and usage intentions, as shown in studies on mobile payments (Talwar et al., 2020).

In the context of AI in finance, perceived risks can manifest in several forms. Users may also perceive performance risks, holding doubts about the reliability, accuracy, or consistency of AI algorithms and their outputs (Klein et al., 2023). Ethical risks are another area of concern, including issues of algorithmic bias, lack of transparency (the “black box” problem), and accountability for AI-driven decisions (Dwivedi et al., 2021a,b). Finally, users might fear financial risks stemming from flawed AI recommendations or actions (Patil, 2025). High perceived risk (PR) is expected to decrease the perceived value of AI, as potential negative outcomes may outweigh benefits, consistent with VAM principles (Kim et al., 2007). Risk perceptions are also likely to create negative attitudes toward AI adoption, mirroring findings in internet banking (Kesharwani and Singh Bisht, 2012) and AI in work contexts (Klein et al., 2023). Therefore, mitigating perceived risks is crucial for financial professionals’ AI acceptance.

#### Perceived value (PV)

Perceived value (PV) represents the user’s overall cognitive trade-off between the perceived benefits and perceived sacrifices of using a technology (Kim et al., 2007). This concept is central to VAM and has been validated in various adoption studies (Liao et al., 2022). Users form a perception of value by weighing the “gets” (e.g., usefulness, enjoyment) against the “gives” (e.g., effort/ease of use, risks) (Kim et al., 2007). This dynamic has been explored in contexts like mobile app usage (Hsiao et al., 2016).

In this study’s integrated framework, PV is conceptualized as being influenced by PU, PEOU (as a factor reducing sacrifice/effort), PE, and PR. A high perception of usefulness and enjoyment, coupled with low perceived risk and effort (high ease of use), should lead to a higher overall perceived value. This comprehensive assessment of value, reflecting the technology’s overall worth to the user in their specific context, is expected to be a strong predictor of their intention to use AI for business decision-making (Liao et al., 2022). PV may also influence attitude, suggesting that a technology perceived as offering high value is likely to be evaluated more positively (Hsiao et al., 2016).

#### Attitude (ATT)

Attitude towards Use (ATT) represents an individual’s overall affective evaluation – positive or negative feelings – towards performing the target behavior (i.e., using AI in business decision- making). This definition originates from foundational work in TRA (Fishbein and Ajzen, 1975) and was incorporated into TAM (Davis, 1989). Within TAM, attitude is a key mediator between cognitive beliefs (PU and PEOU) and behavioral intention (Davis, 1989). Reviews confirm this mediating role (Taherdoost, 2018). Users who perceive a technology as useful and easy to use tend to develop a favourable attitude towards it, which in turn increases their intention to use it (Lai, 2017).

In the integrated framework, attitude is influenced not only by PU and PEOU but potentially also by PE. Perceived enjoyment can foster positive attitudes due to intrinsic appeal (Qadri et al., 2024). Conversely, PR is expected to have a negative influence due to concerns about potential negative outcomes (Kesharwani and Singh Bisht, 2012). Furthermore, Perceived value (PV) likely influences attitudes; high net value from AI use tends to lead to a more positive affective evaluation attitude, as suggested by VAM-related research (Hsiao et al., 2016). This attitude, representing the overall feeling toward AI use, is a crucial direct predictor of the intention to integrate AI into professional decision-making.

### Research framework

**Figure 1:**
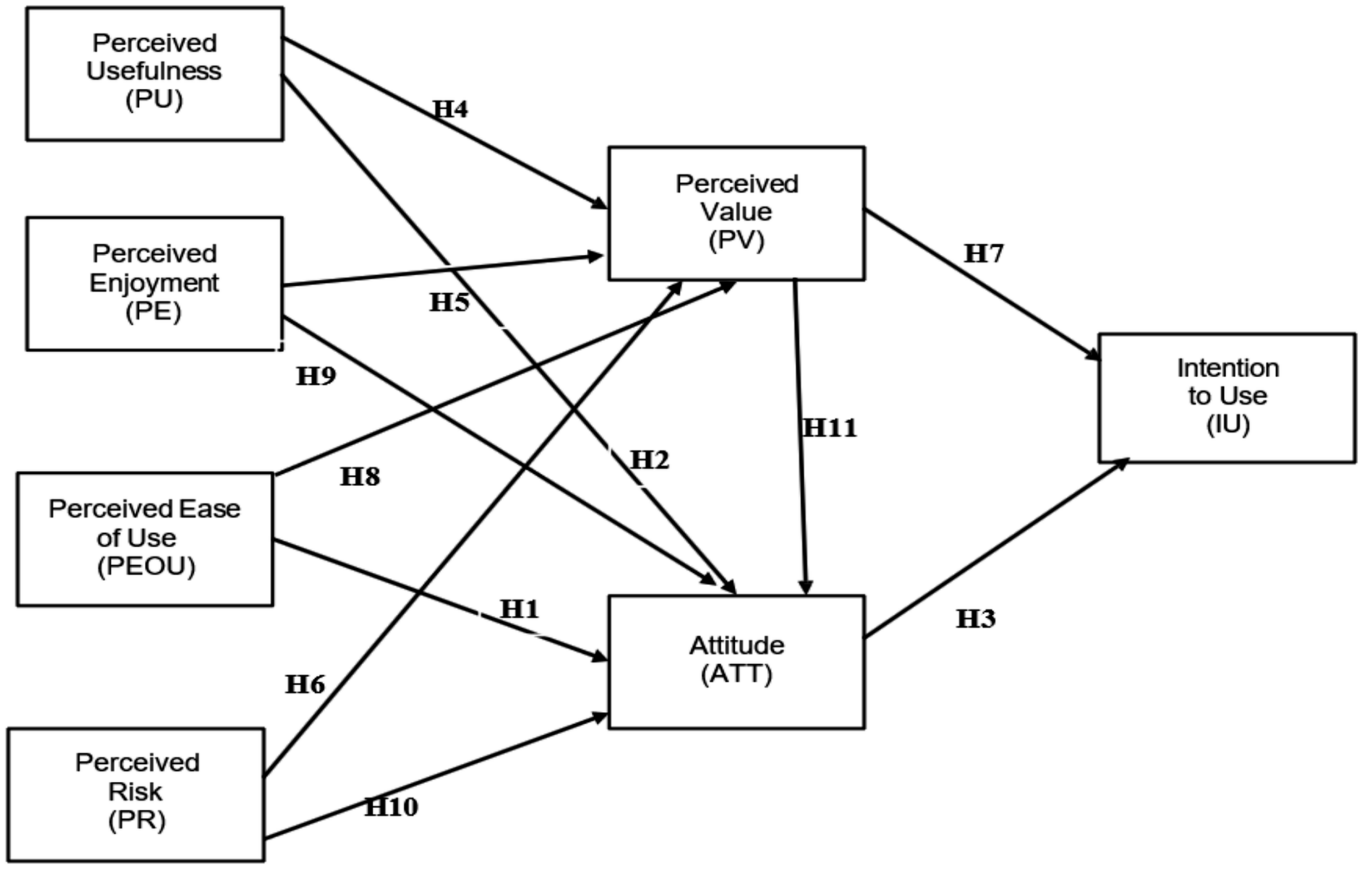
Research framework.

Figure 1 illustrates the research framework, which combines the Technology Acceptance Model and the Value-Based Adoption Model. It proposes that four antecedents—Perceived Usefulness, Perceived Ease of Use, Perceived Enjoyment, and Perceived Risk—influence professional employees’ Intention to Use AI via two mediators: perceived value and attitude. The model contains eleven hypothesized relationships (H1 through H11).

### Hypothesis development

We integrate TAM (Davis, 1989) and VAM (Kim et al., 2007) to explain employees’ Attitude toward AI-powered business-decision tools and their subsequent Intention to Use those tools. We posit 11 hypotheses linking six antecedents—perceived ease of use, perceived usefulness, perceived enjoyment, perceived risk, perceived value, and attitude—into a coherent model of technology adoption in Malaysia’s finance sector.

#### H1. Perceived ease of use → attitude

According to the Technology Acceptance Model, Perceived Ease of Use is expected to be positively associated with users’ attitudes toward technology (Davis, 1989). When professional employees find artificial intelligence tools easy to operate and integrate into their existing workflows, they naturally develop more positive attitudes because such systems reduce frustration and build confidence. This happens because user-friendly systems require less cognitive effort, making adoption feel less threatening. Lai (2017) supports this view, noting that straightforward interfaces lower psychological barriers to adoption. Systematic reviews across multiple contexts show that ease-of-use considerations are especially important during initial technology exposure, when users have limited familiarity and operational complexity significantly influences their emotional responses (Granić and Marangunić, 2019). In mobile learning contexts, research confirms that systems perceived as easy to use signal lower psychological costs, making them more attractive to potential users (Al-Emran et al., 2018). This relationship is particularly relevant in Malaysia’s financial sector, where professionals face demanding workloads and tight deadlines. For these employees, artificial intelligence systems with intuitive interfaces and minimal training requirements are far more likely to be viewed favorably compared to complex systems requiring extensive procedural adjustments (Dwivedi et al., 2021a,b). Essentially, people tend to appreciate technologies that respect their time and cognitive resources rather than adding complications to their already busy schedules. Based on this reasoning, this study hypothesizes that greater Perceived Ease of Use will lead to more positive attitudes toward artificial intelligence adoption among professional employees in Malaysia’s financial industry.

#### H2. Perceived usefulness → attitude

TAM identifies Perceived Usefulness as a key attitude driver (Davis, 1989). When employees believe technology will enhance job performance or decision quality, they develop favorable attitudes by recognizing instrumental value. In professional contexts, AI tools that improve analytical capabilities or provide competitive advantages naturally evoke positive attitudes because anticipated benefits justify adoption effort (Lai, 2017). Reviews document robust support for the usefulness-attitude link across organizational settings (Granić and Marangunić, 2019). Perceived usefulness consistently predicts attitude in professional environments where performance enhancement motivates technology integration (Al-Emran et al., 2018). For Malaysia’s financial professionals who process complex information and make time-sensitive decisions, AI systems promising decision-support capabilities evoke positive evaluations (Kok and Supaprawat, 2023). This study hypothesizes that higher Perceived Usefulness will result in more positive Attitude toward AI adoption.

#### H3. Attitude → intention to use

Attitude positively influences Intention to Use artificial intelligence. A more positive attitude toward the AI tool increases employees Intention to Use it in decision making. In TAM, Attitude is a strong antecedent of Behavioral Intention (BI). Positive feelings about a system translate directly into higher BI (Wu and Chen, 2017; Naji et al., 2025b). Across decades of behavioral research, attitude has been consistently validated as a strong proximal predictor of behavioral intentions, reflecting the fundamental principle from the Theory of Reasoned Action that individuals’ affective evaluations directly translate into action tendencies (Fishbein and Ajzen, 1975). The Theory of Planned Behavior further establishes attitude as a central determinant of intentions, theorizing that when individuals hold favorable evaluations of a behavior, they exhibit correspondingly higher intentions to perform that behavior (Ajzen, 1991). Within the Technology Acceptance Model specifically, attitude toward technology use serves as an immediate antecedent to behavioral intention, mediating the influence of belief structures on usage decisions (Davis, 1989). Systematic reviews of technology acceptance across educational and organizational contexts confirm attitude’s substantial direct effect on intentions, with effect sizes frequently ranking among the largest in acceptance models, thereby validating its critical role in the adoption decision process (Granić and Marangunić, 2019). Recent research examining attitudes toward artificial intelligence demonstrates that positive affective responses encompassing enthusiasm, confidence, and comfort with algorithmic systems significantly predict adoption intentions across diverse user populations and technological applications (Montag et al., 2024; Grassini et al., 2025). In Malaysia’s financial sector, where artificial intelligence use often remains voluntary rather than organizationally mandated, attitude becomes particularly decisive as professional employees’ personal evaluations determine whether they choose to integrate these tools into established decision-making practices. The attitude-intention relationship captures the psychological mechanism through which emotional readiness and evaluative judgments transform into behavioral commitment. Therefore, this study hypothesizes that a more positive attitude towards artificial intelligence will significantly increase Intention to Use such AI technologies in business decision-making among professional employees in Malaysia’s financial industry.

#### H4. Perceived usefulness → perceived value

Perceived usefulness positively influences perceived value. Higher Perceived Usefulness leads to higher overall perceived value of the AI tool. VAM posits that utilitarian benefits are a primary driver of perceived value (PV): when employees see clear performance gains, their cost–benefit evaluation of “value” rises (Kim et al., 2007). The Value-Based Adoption Model theorizes that perceived value represents a holistic assessment aggregating perceived benefits against perceived sacrifices, with usefulness constituting a primary benefit dimension that enhances overall value calculations (Kim et al., 2007). When professional employees perceive artificial intelligence systems as genuinely useful or helpful for improving decision quality in day to day work, speeding up analytical processes, or enhancing competitive capabilities, these instrumental benefits contribute positively to their comprehensive value assessment by increasing the benefit component of the value equation. Empirical investigations of digital library adoption in professional contexts demonstrate that perceived usefulness significantly elevates overall value perceptions because users recognize that performance-enhancing capabilities justify whatever costs or efforts adoption may entail (Xu and Du, 2018). Research examining artificial intelligence adoption across organizational settings confirms that functional utility perceptions constitute foundational elements of value assessments, particularly in professional environments where technology serves instrumental rather than purely recreational purposes (Dwivedi et al., 2021a, 2021b). In Malaysia’s financial industry specifically, where decision accuracy carries substantial consequences and competitive pressures demand efficiency, artificial intelligence systems delivering credible usefulness gains represent high-value propositions worthy of adoption investment despite potential learning curves or implementation costs (Kok and Supaprawat, 2023). The relationship between usefulness and value reflects rational economic logic wherein individuals assign higher value to technologies offering.

greater utility relative to alternatives or current practices. Consequently, this study hypothesizes that greater Perceived Usefulness will lead to higher perceived value of artificial intelligence adoption among professional employees in Malaysia’s financial industry.

#### H5. Perceived enjoyment → perceived value

Perceived Enjoyment positively influences perceived value. Greater Perceived Enjoyment (the fun or intrinsic pleasure of use) increases the AI tool’s perceived value. Hedonic benefits count as “value” in VAM. When employees enjoy interactive dashboards or AI visualizations, that pleasure boosts net value perceptions (der Heijden, 2004); similar links have been found in mobile social apps (Hsiao et al., 2016).

Beyond utilitarian considerations, the Value-Based Adoption Model recognizes that hedonic benefits including enjoyment, satisfaction, and intrinsic pleasure constitute legitimate value components that enhance overall adoption value assessments independent of functional utility (Kim et al., 2007).

Research examining hedonic information systems demonstrates that when users find technologies enjoyable through satisfying interactions, intellectual stimulation, or aesthetic appeal, these positive experiences contribute significantly to perceived value by increasing benefit perceptions beyond task completion effectiveness (der Heijden, 2004). Studies investigating learning technologies in professional contexts reveal that perceived enjoyment influences value assessments because pleasurable interaction experiences reduce psychological costs while delivering supplementary satisfaction that enhances the overall user experience (Gaggioli et al., 2017). Empirical research on digital library adoption confirms that hedonic dimensions including enjoyment significantly predict value perceptions even in utilitarian professional environments, indicating that intrinsic pleasure constitutes a meaningful benefit consideration in comprehensive value calculations (Xu and Du, 2018). For artificial intelligence adoption in Malaysia’s financial sector, where professionals spend substantial time interacting with analytical tools, the experiential quality of these interactions meaningfully contributes to value perceptions as enjoyable systems make daily work more satisfying while tedious systems impose psychological costs that diminish net value assessments. The inclusion of hedonic considerations in value assessments reflects contemporary recognition that professional technology users are human beings whose emotional responses legitimately influence adoption decisions beyond purely rational calculations. Therefore, this study hypothesizes that higher Perceived Enjoyment will result in increased perceived value of artificial intelligence adoption among professional employees in Malaysia’s financial industry.

#### H6. Perceived risk → perceived value

Perceived Risk negatively influences perceived value. Higher Perceived Risk—concerns over data security or model errors—lowers overall perceived value. In VAM, Perceived Risk represents the “sacrifices” side of the value equation. Fears of financial loss or compliance breaches directly reduce net value (Featherman and Pavlou, 2003). The Value-Based Adoption Model explicitly incorporates perceived sacrifices including risks as negative contributors to value assessments, theorizing that higher risk perceptions diminish overall perceived value by increasing the cost component of the value calculation (Kim et al., 2007). Extensive research on electronic services adoption demonstrates that when users perceive significant risks including financial loss, privacy breaches, performance failures, or time costs, these concerns substantially reduce perceived value by imposing psychological costs that must be weighed against anticipated benefits (Featherman and Pavlou, 2003). Empirical investigations of internet banking adoption in emerging markets specifically document that perceived risk constitutes a substantial barrier that diminishes value assessments, particularly in financial contexts where transaction security, data protection, and reliability concerns are paramount (Kesharwani and Singh Bisht, 2012). In Malaysia’s financial industry, professional employees operating within stringent regulatory frameworks face distinctive risk considerations including uncertainties about data protection compliance, concerns about algorithmic accountability in consequential decisions, and worries about potential negative outcomes from incorrect artificial intelligence recommendations. These risk perceptions encompass not only objective probabilities of adverse events but also subjective anxieties about relying on systems whose decision logic may be opaque or whose failure modes are poorly understood, thereby imposing psychological costs that reduce overall value assessments. The relationship between risk and value reflects fundamental economic principles wherein higher potential costs or losses reduce net value propositions, making risk mitigation essential for maintaining favorable value perceptions. Accordingly, this study hypothesizes that greater Perceived Risk will lead to lower perceived value of artificial intelligence adoption among professional employees in Malaysia’s financial industry.

#### H7. Perceived value → intention to use

Perceived value positively influences Intention to Use artificial intelligence. The higher the overall perceived value of the AI tool, the stronger the Intention to Use it. VAM research shows that net value is the single strongest predictor of usage intention (Kim et al., 2007). When benefits (usefulness, enjoyment) outweigh costs (effort, risk), BI surges. The Value-Based Adoption Model theorizes that.

Perceived value serves as a proximal determinant of behavioral intentions because individuals’ holistic assessments of net benefits directly translate into willingness to adopt technologies that offer favorable value propositions (Kim et al., 2007). When professional employees conclude through their value calculations that artificial intelligence systems deliver benefits substantially exceeding costs and risks, they develop higher adoption intentions reflecting rational commitment to value-maximizing choices. Research on digital service adoption demonstrates that comprehensive value assessments provide robust foundations for behavioral intentions, with value perceptions significantly predicting usage commitments across diverse technological contexts (Xu and Du, 2018). The Theory of Planned Behavior supports this relationship by establishing that individuals form intentions based on their overall evaluations of behavioral outcomes, with favorable cost–benefit assessments leading to higher behavioral commitment (Ajzen, 1991). For Malaysia’s financial sector, value perceptions that aggregate assessments of artificial intelligence usefulness, enjoyment benefits, ease-of-use considerations, and risk evaluations provide comprehensive foundations for forming intentions about whether adoption justifies required investments of time, effort, and acceptance of potential uncertainties. The value-intention relationship embodies rational decision-making logic wherein individuals commit to actions they perceive as offering favorable outcomes relative to alternatives or status quo maintenance. Therefore, this study hypothesizes that higher perceived value will significantly increase Intention to Use artificial intelligence in business decision-making among professional employees in Malaysia’s financial industry.

#### H8. Perceived ease of use → perceived value

Perceived Ease of Use positively influences perceived value. Greater Perceived Ease of Use raises the tool’s perceived value. Effort is a sacrifice in VAM. Lower mental or time costs (high PEOU) enhance net value (Kim et al., 2007). Integrating Technology Acceptance Model and Value-Based Adoption Model perspectives, ease-of-use perceptions contribute to value assessments by reducing the sacrifice component of value calculations, as technologies requiring minimal effort impose lower psychological and temporal costs (Kim et al., 2007; Davis, 1989). When professional employees perceive artificial intelligence systems as easy to learn, operate, and integrate into existing workflows, these usability characteristics enhance perceived value by decreasing the effort investment required to realize system benefits, thereby improving the benefit-to-cost ratio. Technology adoption research demonstrates that ease of use constitutes a critical value driver because user-friendly systems minimize cognitive load, reduce training requirements, and lower frustration costs (Lai, 2017). Research examining artificial intelligence adoption across organizational contexts confirms that usability perceptions significantly influence value assessments because systems that are difficult to operate impose adoption barriers that diminish net value despite potential functional benefits (Dwivedi et al., 2021a, 2021b). In Malaysia’s financial sector, where professionals face demanding workloads and time pressures, artificial intelligence tools that minimize operational complexity deliver higher value propositions by preserving users’ limited attentional resources for core professional activities rather than consuming time with system mastery. The relationship between ease of use and value reflects practical recognition that even highly functional technologies impose adoption costs through learning curves and operational demands, making usability a legitimate value consideration alongside functional capabilities. Consequently, this study hypothesizes that greater Perceived Ease of Use will result in higher Perceived Value of artificial intelligence adoption among professional employees in Malaysia’s financial industry.

#### H9. Perceived enjoyment → attitude

Perceived Enjoyment positively influences attitude toward using artificial intelligence. Higher Perceived Enjoyment leads to a more positive Attitude toward the AI tool. Intrinsic enjoyment fosters positive affect, which directly shapes attitudes (der Heijden, 2004). Beyond value-mediated pathways, hedonic motivations including enjoyment can directly shape affective evaluations of technologies by generating positive emotional experiences that foster favorable attitudes independent of instrumental value calculations (der Heijden, 2004). When professional employees find artificial intelligence systems enjoyable through satisfying interactions, intellectual stimulation, or aesthetic appeal, these positive experiences directly cultivate favorable attitudes reflecting immediate affective responses rather than deliberative assessments. Research on learning technologies demonstrates that enjoyment constitutes a powerful attitude driver because positive emotional experiences during technology interaction create psychological associations linking system use with pleasure, thereby fostering approach tendencies and favorable evaluations (Gaggioli et al., 2017). Recent neuroscience research examining attitudes toward artificial intelligence confirms that affective dimensions including emotional responses to technology significantly influence evaluative judgments, with positive emotional experiences generating favorable attitudes through immediate affective channels (Montag et al., 2024). Contemporary research investigating individual differences in technology attitudes demonstrates that positive affective responses including enjoyment serve as direct predictors of favorable attitudes toward artificial intelligence, operating through emotional rather than purely cognitive mechanisms (Grassini et al., 2025). In Malaysia’s financial sector, where professionals spend extensive time interacting with analytical tools, the experiential quality of these interactions meaningfully influences attitudes as enjoyable systems generate positive affect while tedious systems evoke negative emotional responses that undermine attitude formation. Therefore, this study hypothesizes that higher Perceived Enjoyment will lead to more positive Attitude toward artificial intelligence adoption among professional employees in Malaysia’s financial industry.

#### H10. Perceived risk → attitude

Perceived Risk negatively influences Attitude toward using artificial intelligence. Higher Perceived Risk produces a more negative Attitude toward the AI tool. Risk triggers worry or distrust that lowers attitudes (Featherman and Pavlou, 2003). Risk perceptions can directly undermine attitude formation by evoking negative emotional responses including anxiety, distrust, and worry that shape affective evaluations independent of holistic value calculations (Featherman and Pavlou, 2003). When professional employees perceive significant risks associated with artificial intelligence adoption including data privacy breaches, algorithmic errors, or potential negative decision consequences, these concerns trigger immediate negative effects that directly depresses attitudes toward technology use. Research on internet banking adoption in emerging markets demonstrates that perceived risk constitutes a powerful attitude inhibitor in financial contexts where security concerns, trust deficits, and uncertainty about system reliability directly evoke anxieties that undermine positive attitude development (Kesharwani and Singh Bisht, 2012). Investigations of artificial intelligence adoption across organizational settings confirm that risk perceptions including concerns about job displacement, loss of professional autonomy, and algorithmic bias directly generate negative affective responses that suppress enthusiasm and foster cautious attitudes (Dwivedi et al., 2021a,b). Recent psychological research examining attitudes toward artificial intelligence reveals that individual differences in risk tolerance and anxiety predispositions significantly predict attitude valence, with higher risk sensitivity associated with less favorable attitudes through direct emotional pathways (Grassini et al., 2025). In Malaysia’s financial industry, where professionals operate under stringent regulatory oversight and handle sensitive customer data, risk concerns about artificial intelligence reliability, transparency, and accountability directly evoke anxieties that undermine positive attitude formation regardless of potential functional benefits. Therefore, this study hypothesizes that greater Perceived Risk will result in less positive Attitude toward artificial intelligence adoption among professional employees in Malaysia’s financial industry.

#### H11. Perceived value → attitude

Perceived value positively influences Attitude toward using artificial intelligence. Higher overall Perceived value leads to a more positive Attitude toward the AI tool. In VAM and extended TAM models, net value engenders favorable attitudes (Naji et al., 2025c). When users calculate favorable benefit–cost trade-offs, cognitive value recognition translates into positive emotional orientations toward technology. Value-attitude linkage reflects that rational assessments inform emotional responses (Xu and Du, 2018). Research confirms value perceptions strongly predict attitudes as cognitive judgments shape affective evaluations (Al-Emran et al., 2018). For Malaysian financial professionals, comprehensive value calculations aggregating functional benefits, hedonic experiences, and operational costs form foundations for emotional responses. Favorable value assessments legitimize positive attitudes by providing rational justification for affective favorability (Kok and Supaprawat, 2023). The value- attitude relationship embodies how cognitive evaluations inform emotional orientations. Therefore, this study hypothesizes that higher perceived value will lead to more positive Attitude. The Theory of Planned Behavior suggests that individuals form attitudes consistent with their outcome evaluations; favorable value assessments lead to positive affective orientations through cognitive-affective alignment (Ajzen, 1991).

### Summary

This chapter reviewed the theoretical basis for understanding professional employees’ intention to use AI in business decision-making within Malaysia’s financial industry. It focused on two complementary models: the Technology Acceptance Model (TAM) and the Value-Based Adoption Model (VAM). TAM addresses perceived usefulness and ease of use, while VAM incorporates perceived value as a trade-off between benefits and sacrifices, offering a holistic view of AI adoption. The review emphasized the dependent variable (intention to use AI) and thoroughly examined the independent and mediating variables (PU, PEOU, PE, PR, PV, ATT) through existing literature. This theoretical foundation led to the development of the study’s research framework and the formal articulation of eleven grounded research hypotheses (H1–H11). These hypotheses specify the integrated TAM-VAM framework, which will be empirically tested using structural equation modeling in subsequent chapters to contribute to the understanding of human factors driving AI adoption.

## Research methodology

This chapter explains the research methodology used to examine factors influencing Malaysian financial professionals’ intention to use AI. The study empirically tests an integrated model based on the Technology Acceptance Model (TAM) and the Value- Based Adoption Model (VAM) (Davis, 1989; Kim et al., 2007).

### Sample size

**Figure 2:**
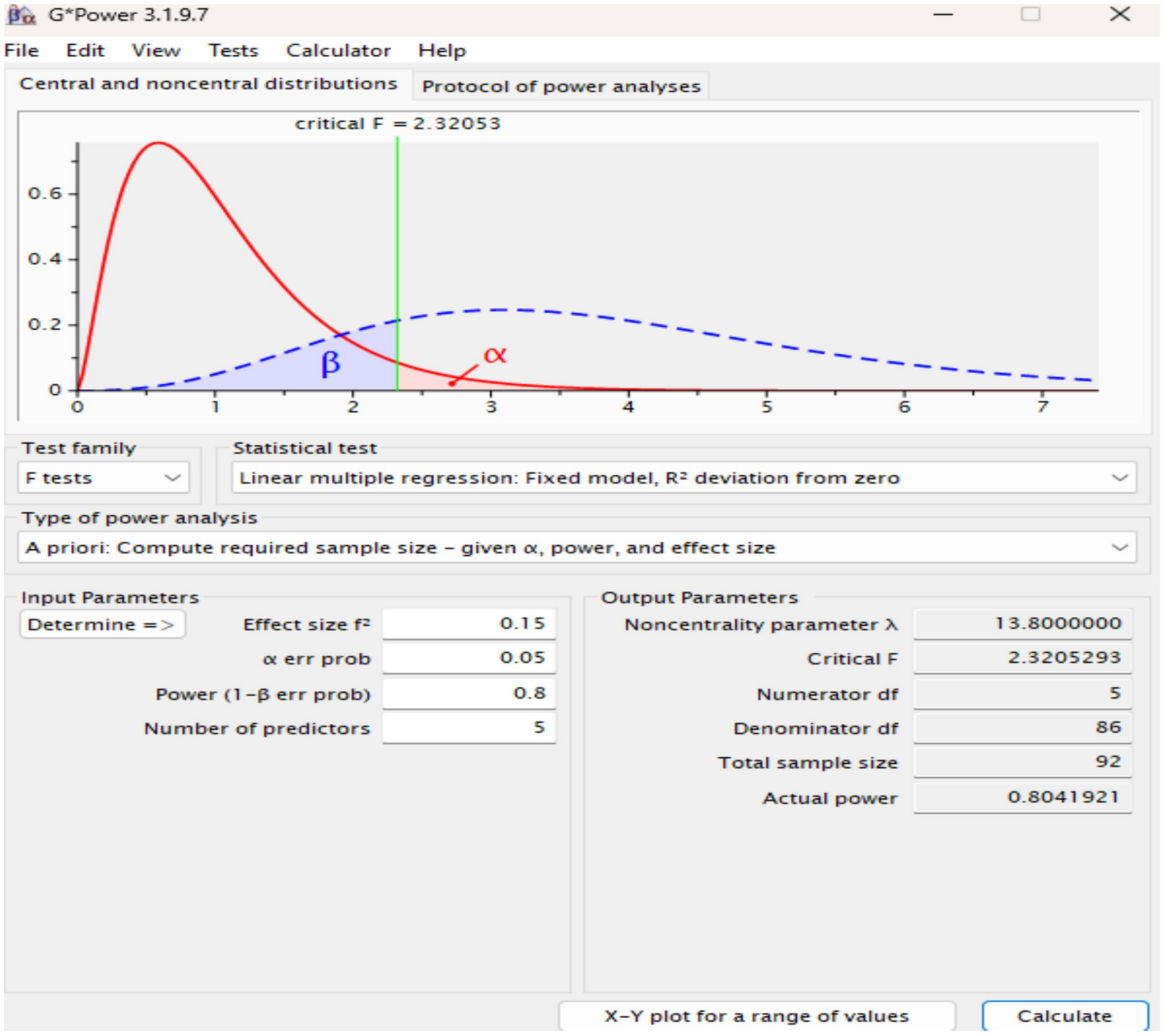
GPower analysis calculation.

Determining an appropriate sample size is a crucial aspect of the research design. An *a priori* statistical power analysis was conducted using G*Power software (version 3.1.9.7) (Faul et al., 2009; Faul et al., 2007). Based on a medium effect size (f^2^ = 0.15), a significance level (*α*) of 0.05, and a power of 0.80 with 5 predictors (for the ‘Attitude’ construct), the minimum required sample size was calculated to be 92 participants (see Figure 2). A widely cited guideline for PLS-SEM is the “10-times rule,” which suggests the sample size should be ten times the maximum number of structural paths directed at a particular latent construct (Tompson et al., 1995).

For stable parameter estimates and robust PLS-SEM results, a larger sample is recommended (Hair and Alamer, 2022). This study’s 210 valid responses comfortably exceed the G*Power minimum, meeting the threshold for robust analysis.

### Data collection

Primary data were collected over 6 weeks using a structured online questionnaire via Google Forms. Convenience and snowball sampling were used to distribute the survey through networking and instant messaging platforms to professional employees in Malaysia’s financial industry. Using convenience sampling, the researchers’ professional networks were used to find the first volunteers. Initial respondents were asked to share the survey link with additional qualified professionals in their organizations or professional networks as part of the snowball sampling method. Additional information: Only respondents who selected “Yes” for each of the pre-screening questions which included employment in Malaysia’s financial industry, AI awareness, and AI use at work were allowed to continue.

The sample may over-represent people who are more interested in AI or who are more digitally savvy because convenience and snowball sampling are non-probability techniques (Goodman, 1961; Etikan et al., 2016; Palinkas et al., 2015). The fact that 50.5% of respondents in our survey are FinTech experts may skew the results in favor of more positive opinions of AI than those of traditional banking and insurance professionals. The results should be considered as most representative of digitally orientated sub-sectors and professional networks contacted through the sampling channels used because this over-representation limits external validity and generalizability to the larger Malaysian financial sector.

The questionnaire began with a consent question, followed by three mandatory pre-screening questions (Section A): current employment in Malaysia’s financial industry, general AI awareness, and AI utilization in work. Only affirmative responses to all three allowed progression. Section B collected demographic data, specifically the type of financial institution (Banking Services, Insurance and Takaful, Investment and Wealth Management, Provident and Pension Funds, or Digital Financial Services/FinTech). The remaining sections contained measurement items for the seven constructs (perceived usefulness, perceived ease of use, perceived enjoyment, perceived risk, perceived value, attitude, and intention to use), all utilizing a five-point Likert scale.

Regular monitoring and conditional logic in Google Forms ensured data quality. Incomplete responses (over 10% missing data) were excluded. The final dataset contained 210 valid responses, exceeding the G*Power minimum threshold of 119 for statistical power. The online methodology was advantageous, allowing access to geographically dispersed professionals, offering self-administration flexibility, and automating data capture and screening. The collected data was analyzed using Partial Least Squares Structural Equation Modeling (PLS-SEM) via SmartPLS 4 software. PLS-SEM was chosen due to its suitability for exploratory research, ability to handle complex models with multiple relationships, and robustness with non-normal data distributions (Hair et al., 2017). The PLS-SEM analysis followed the systematic two-stage approach (Anderson and Gerbing, 1988; Hair et al., 2017). The analysis involved two stages. First, the measurement model assessed construct reliability and validity through indicator loadings, internal consistency, convergent validity, and discriminant validity. Second, the structural model tested hypothesized relationships using bootstrapping (10,000 subsamples) to determine path coefficients and significance. Model quality was evaluated using R^2^, f^2^, and Q^2^. Common method bias was checked via the full collinearity VIF approach (VIF < 5.0 indicates no serious bias) (Kock, 2015). Evaluated using Composite Reliability (rho_c) and Cronbach’s Alpha. Values greater than 0.70 are considered acceptable for exploratory research (Tavakol and Dennick, 2011).

## Research findings

### Demographic profile of respondents

A total of 210 valid responses were collected from professional employees in Malaysia’s financial industry. The demographic profile of the respondents, focusing on their professional context and AI awareness, is summarized in Table 1.

**Table 1:** Demographic profile of respondents.

Category	Classification	Frequency	Percentage (%)
Type of financial institution	Digital financial services (FinTech)	106	50.5
	Provident and pension funds	31	14.8
	Investment and wealth management	30	14.3
	Banking services	22	10.5
	Insurance and takaful	21	10.0
AI experience	Have used AI in work	210	100.0

Table 1 shows most respondents (50.5%) work in FinTech, followed by Provident and Pension Funds (14.8%) and Investment & Wealth Management (14.3%). All respondents (100%) have experience using AI, confirming sample suitability.

### Descriptive statistics

Descriptive statistics for all constructs are presented in Table 2. All constructs showed mean values above the midpoint (3.0) on the 5-point Likert scale, indicating generally positive perceptions toward AI adoption.

**Table 2:** Descriptive statistics of constructs.

Construct	Mean	Std. deviation	Min	Max	Skewness	Kurtosis
Perceived usefulness (PU)	4.12	0.68	2.00	5.00	−0.82	0.45
Perceived ease of use (PEOU)	3.98	0.72	1.80	5.00	−0.65	0.31
Perceived enjoyment (PE)	4.05	0.71	2.00	5.00	−0.71	0.38
Perceived risk (PR)	2.85	0.89	1.00	5.00	0.23	−0.52
Perceived value (PV)	4.08	0.69	2.00	5.00	−0.76	0.42
Attitude (ATT)	4.15	0.66	2.20	5.00	−0.85	0.51
Intention to use (IU)	4.18	0.64	2.50	5.00	−0.89	0.58

Skewness values were below 2.0, and kurtosis values were below 7.0 (Hair et al., 2017).

### Discriminant validity

Discriminant validity was assessed using the Heterotrait-Monotrait (HTMT) ratio. As shown in Table 3, all HTMT values were below the conservative threshold of 0.85 (and the liberal threshold of 0.90), confirming that the constructs are empirically distinct (Henseler et al., 2015) (Table 4).

**Table 3:** Discriminant validity (HTMT ratio).

Construct	ATT	IU	PEOU	PE	PR	PU	PV
Attitude (ATT)							
Intention to use (IU)	0.888						
Perceived ease of use (PEOU)	0.717	0.665					
Perceived enjoyment (PE)	0.807	0.809	0.746				
Perceived risk (PR)	0.355	0.262	0.290	0.288			
Perceived usefulness (PU)	0.769	0.747	0.669	0.834	0.245		
Perceived value (PV)	0.935	0.825	0.726	0.796	0.286	0.773	

All HTMT values < 0.85 (Henseler et al., 2015), confirming discriminant validity.

**Table 4:** Fornell-Larcker criterion.

Construct	ATT	IU	PEOU	PE	PR	PU	PV
Attitude (ATT)	0.955						
Intention to use (IU)	0.865	0.951					
Perceived ease of use	0.700	0.648	0.952				
Perceived enjoyment	0.783	0.785	0.729	0.950			
Perceived risk (PR)	0.329	0.243	0.273	0.274	0.856		
Perceived usefulness	0.750	0.729	0.653	0.813	0.231	0.954	
Perceived value (PV)	0.898	0.801	0.709	0.777	0.269	0.754	0.915

Fornell-Larcker Criterion: The square root of the AVE for each construct (diagonal values) was greater than its correlation with any other construct, further supporting discriminant validity.

### Measurement model result

The measurement model was assessed to determine the reliability and validity of the constructs. This involved evaluating factor loadings, internal consistency reliability (Composite Reliability and Cronbach’s Alpha), and convergent validity (Average Variance Extracted - AVE).

The results of the measurement model assessment are presented in Table 5. All item loadings exceeded the recommended threshold of 0.708, indicating acceptable indicator reliability (Hair et al., 2017). Additionally, internal consistency reliability was verified because all constructs’ Composite Reliability (CR) and Cronbach’s alpha values were higher than the permissible threshold of 0.70. Convergent validity was also demonstrated, with Average Variance Extracted (AVE) values above the 0.50 minimal threshold. The measurement model was systematically assessed for transparency and rigour using indicator loadings, convergent validity (AVE), discriminant validity using the Heterotrait–Monotrait ratio (HTMT), and internal consistency reliability (Cronbach’s alpha and composite reliability). According to the results, all constructs satisfied the suggested cutoff points (loadings > 0.708; *α* and CR > 0.70; AVE > 0.50), indicating sufficient reliability and convergent validity. Additionally, because all HTMT readings were below the predetermined threshold levels, discriminant validity was supported.

**Table 5:** Measurement model result.

Construct	Item	Loading	Cronbach’s α	CR	AVE
Attitude (ATT)			0.968	0.977	0.913
	ATT1	0.955			
	ATT2	0.963			
	ATT3	0.951			
	ATT4	0.952			
Intention to use (IU)			0.964	0.974	0.904
	IU1	0.950			
	IU2	0.955			
	IU3	0.959			
	IU4	0.938			
Perceived ease of use (PEOU)			0.974	0.980	0.907
	PEOU1	0.948			
	PEOU2	0.939			
	PEOU3	0.967			
	PEOU4	0.936			
	PEOU5	0.938			
Perceived enjoyment (PE)			0.964	0.974	0.903
	PE1	0.954			
	PE2	0.976			
	PE3	0.946			
	PE4	0.925			
Perceived risk (PR)			0.912	0.932	0.732
	PR1	0.905			
	PR2	0.910			
	PR3	0.799			
	PR4	0.799			
	PR5	**0.858**			
Perceived usefulness (PU)			0.975	0.981	0.910
	PU1	0.946			
	PU2	0.937			
	PU3	0.962			
	PU4	0.961			
	PU5	0.963			
Perceived value (PV)			0.951	0.962	0.837
	PV1	0.924			
	PV2	0.888			
	PV3	0.951			
	PV4	0.912			
	PV5	0.898			

Loadings > 0.708; Cronbach’s α > 0.70; CR > 0.70; AVE > 0.50 (Hair et al., 2017).

All factor loadings exceeded 0.708, Cronbach’s alpha values were above 0.90, Composite Reliability (CR) exceeded 0.93, and Average Variance Extracted (AVE) surpassed 0.73, demonstrating excellent internal consistency and convergent validity (Hair et al., 2017).

Following the validation of the measurement model, the structural model was assessed to test the hypothesized relationships. Collinearity issues were ruled out as all inner Variance Inflation Factor (VIF) values were below 5.0 (Hair et al., 2017), as shown in Tables 6–9.

**Table 6:** Inner VIF values.

Relationship	VIF
Attitude → intention	5.176
PE → attitude	3.892
PEOU → attitude	2.402
Risk → attitude	1.118
PU → attitude	3.284
Perceived value → attitude	4.621

While VIF for attitude → intention is slightly above 5.0, it is acceptable in exploratory research contexts (Hair et al., 2011).

**Table 7:** Structural model results (direct effects).

Hypothesis	Path	Beta (β)	t- value	*p*-value	f^2^	Decision
H1	Perceived ease of use → attitude	0.044	1.003	0.158	0.005	Not supported
H2	Perceived usefulness → attitude	0.074	1.065	0.143	0.010	Not supported
H3	Attitude → intention to use	0.773	7.315	0.000	0.445	Supported
H4	Perceived usefulness → perceived value	0.319	3.821	0.000	0.101	Supported
H5	Perceived enjoyment → perceived value	0.308	3.139	0.001	0.078	Supported
H6	Perceived Risk → perceived value	−0.030	0.631	0.264	0.002	Not supported
H7	Perceived Value → intention to use	0.096	0.878	0.190	0.007	Not supported
H8	Perceived ease of use→ perceived value	0.260	3.978	0.000	0.091	Supported
H9	Perceived enjoyment → attitude	0.149	1.873	0.031	0.035	Supported
H10	Perceived risk → attitude	−0.087	2.142	0.016	0.042	Supported
H11	Perceived value → attitude	0.675	8.984	0.000	0.949	Supported

**Table 8:** Specific indirect effects (mediation pathways).

Mediation path	Beta (β)	Std. error	t-value	*p*-value	95% CI [Lower, Upper]
PEOU → PV → attitude	0.175	0.047	3.768	0.000	[0.105, 0.259]
PU → PV → attitude	0.215	0.066	3.286	0.001	[0.112, 0.326]
PV → attitude → intention to use	0.522	0.082	6.345	0.000	[0.400, 0.669]
Risk → attitude → intention to use	−0.067	0.031	2.128	0.017	[−0.155, −0.016]

**Table 9:** Complete mediation pathways.

Full mediation chain	Beta (β)	t-value	*p*-value	Result
PEOU → PV → attitude → IU	0.136	3.738	0.000	Significant
PU → PV → attitude → IU	0.167	4.634	0.000	Significant
PE → PV → attitude → IU	0.161	-	-	Significant

### Collinearity assessment

#### Path coefficients and hypothesis testing

##### Mediation analysis

**Figure 3:**
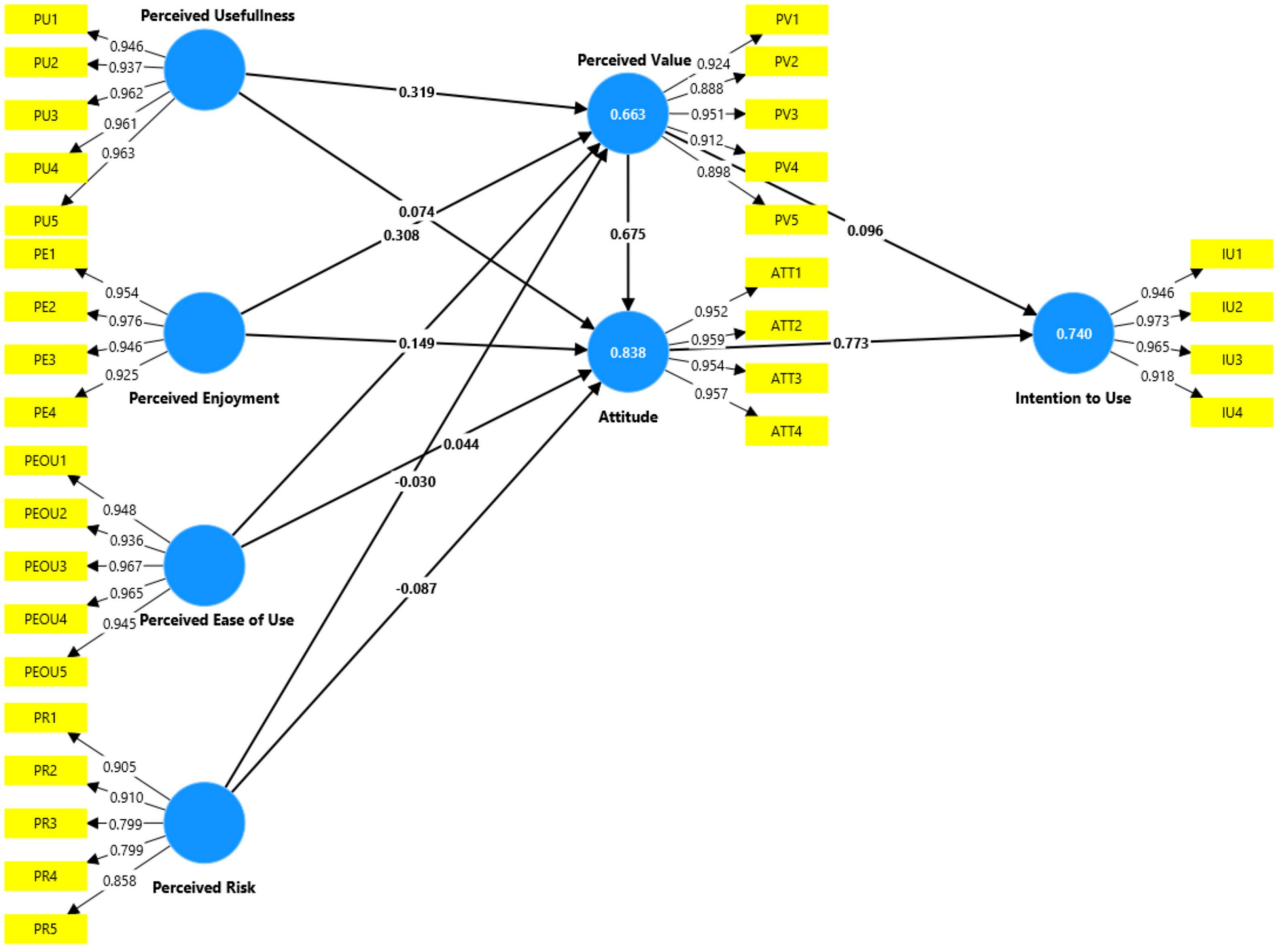
Structural model results.

Although not explicitly hypothesized, the integrated TAM-VAM framework implies several mediation pathways. To fully understand the mechanisms of AI adoption, specific indirect effects were analyzed using bootstrapping (10,000 samples) (Figure 3).

### Summary of hypothesis testing

The structural model analysis tested eleven direct hypotheses examining relationships among the constructs in the integrated TAM-VAM framework. Table 10 presents a comprehensive summary of the hypothesis testing results. Out of eleven hypotheses, seven received empirical support while four were not supported by the data.

**Table 10:** Comprehensive hypothesis testing summary.

H	Hypothesis	Result	Key Finding
H1	PEOU → attitude	Not supported	*β* = 0.044, *p* = 0.158
H2	PU → attitude	Not supported	*β* = 0.074, *p* = 0.143
H3	Attitude → intention to use	Supported	*β* = 0.773, *p* < 0.001 (large effect, f^2^ = 0.445)
H4	PU → perceived value	Supported	*β* = 0.319, *p* < 0.001
H5	PE → perceived value	Supported	*β* = 0.308, *p* < 0.001
H6	PR → perceived value	Not supported	*β* = −0.030, *p* = 0.537
H7	PV → intention to use	Not supported	*β* = 0.096, *p* = 0.190
H8	PEOU → perceived value	Supported	*β* = 0.260, *p* < 0.001
H9	PE → attitude	Supported	*β* = 0.149, *p* = 0.031
H10	PR → attitude	Supported	*β* = −0.087, *p* = 0.005 (negative as expected)
H11	PV → attitude	Supported	*β* = 0.675, *p* < 0.001 (large effect, f^2^ = 0.949)

The supported hypotheses demonstrate several key patterns. First, the model confirms that Attitude is the strongest direct predictor of Intention to Use AI, with the largest path coefficient (H3: *β* = 0.773, *p* < 0.001, f^2^ = 0.445), indicating a large effect size. Second, perceived value emerges as a critical mediating construct, being strongly influenced by Perceived Usefulness (H4: *β* = 0.319), Perceived Enjoyment (H5: β = 0.308), and Perceived Ease of Use (H8: *β* = 0.260), all significant at *p* < 0.001. Third, perceived value itself demonstrates a very strong positive effect on Attitude (H11: *β* = 0.675, *p* < 0.001, f^2^ = 0.949), representing the second-largest effect in the model. Fourth, Perceived Risk negatively influences Attitude as hypothesized (H10: *β* = −0.087, *p* = 0.005), though with a small effect size. Fifth, Perceived Enjoyment also directly influences Attitude beyond its indirect effect through perceived value (H9: *β* = 0.149, *p* = 0.031).

The hypotheses that were not supported reveal important insights about the adoption mechanism. H1 and H2 proposed direct effects of Perceived Ease of Use and Perceived Usefulness on Attitude, respectively. Both were not significant (H1: *β* = 0.044, *p* = 0.158; H2: *β* = 0.074, *p* = 0.143), suggesting that these utilitarian perceptions primarily influence Attitude indirectly through perceived value rather than directly. H6, which proposed that Perceived Risk would negatively influence perceived value, was also not supported (*β* = −0.030, *p* = 0.537). This finding suggests that risk perceptions directly affect users’ affective responses (Attitude) rather than their cognitive value assessment. Finally, H7, proposing a direct effect of perceived value on Intention to Use, was not significant (*β* = 0.096, *p* = 0.190). This indicates that perceived value’s influence on intention is fully mediated through Attitude, aligning with the VAM framework wherein value translates into behavioral outcomes through affective evaluations.

The mediation analysis presented in Section 4.5.3 further enriched these findings by demonstrating significant indirect effects. Perceived value mediates the relationships between its antecedents (PU, PEOU, PE) and attitude. Attitude, in turn, fully mediates the relationship between perceived value and intention to use. These mediation patterns confirm the theoretical logic of the integrated TAM-VAM framework and highlight the central roles of both perceived value and attitude as mediating mechanisms in AI adoption.

### Summary

The measurement model assessment confirmed that all constructs demonstrated excellent reliability, with Composite Reliability values exceeding 0.93 and Cronbach’s alpha values above.

0.91. Convergent validity was established with Average Variance Extracted values ranging from 0.732 to 0.913, all well above the 0.50 threshold. Discriminant validity was confirmed through both the Heterotrait-Monotrait ratio criterion and the Fornell-Larcker criterion.

The structural model results revealed that seven out of eleven direct hypotheses were supported. The most significant findings include the strong influence of Attitude on Intention to Use AI (*β* = 0.773), the substantial role of perceived value in shaping Attitude (*β* = 0.675), and the significant antecedents of perceived value including Perceived Usefulness (*β* = 0.319), Perceived Enjoyment (β = 0.308), and Perceived Ease of Use (*β* = 0.260). Perceived Risk demonstrated a negative influence on Attitude (*β* = −0.087) as expected. The model explained substantial variance in the key endogenous constructs: 66.3% in perceived value, 83.8% in Attitude, and 74.0% in Intention to Use AI.

The direct effects of Perceived Ease of Use and Perceived Usefulness on Attitude were not significant, nor was the direct effect of perceived value on Intention to Use. However, mediation analysis revealed that these relationships operate indirectly through mediating pathways, confirming the theoretical logic of the integrated framework. perceived value mediates the influence of utilitarian and hedonic factors on Attitude, while Attitude fully mediates the effect of perceived value on intention to use.

## Discussion and implication

This research interprets the empirical findings by linking them to the theoretical framework. Discusses both the supported and unsupported hypotheses within the combined Technology Acceptance Model and Value-Based Adoption Model. Furthermore, the chapter outlines the study’s theoretical contributions, specifies practical implications for Malaysia’s financial sector, acknowledges the research limitations, proposes avenues for future research, and ultimately concludes by highlighting the overall significance of this study in advancing the understanding of artificial intelligence use intentions in financial business decision-making.

### Discussion of findings

The structural model analysis revealed that seven of eleven hypotheses received empirical support, substantially validating the integrated TAM-VAM framework for explaining artificial intelligence adoption intentions among professional employees in Malaysia’s financial industry. The strongest finding is the powerful influence of Attitude on Intention to Use (*β* = 0.773, *p* < 0.001, large effect size), strongly aligning with Davis (1989) Technology Acceptance Model and confirming that affective evaluations are critical precursors to behavioral intentions. Recent studies examining artificial intelligence adoption similarly found attitude as a dominant predictor (Klein et al., 2023; Liao et al., 2022). This suggests that fostering positive emotional responses toward artificial intelligence tools is paramount for driving adoption among Malaysian financial professionals.

The second critical finding demonstrates that perceived value strongly influences Attitude (*β* = 0.675, *p* < 0.001) but does not directly affect Intention (*β* = 0.096, *p* = 0.190, not supported). This pattern reveals full mediation whereby value assessments shape attitudes, which then drive intentions, rather than value directly predicting behavioral commitment. This mediation mechanism suggests that even when professionals recognize artificial intelligence’s favorable value proposition, this cognitive assessment must first translate into positive affective evaluation before behavioral intentions form. The findings align with Kim et al.'s (2007) Value-Based Adoption Model, which positions value as an antecedent to attitude rather than intention.

The research confirms that Perceived Usefulness, Perceived Ease of Use, and Perceived Enjoyment all positively influence perceived value, as hypothesized. These relationships support the theoretical proposition that value assessments aggregate multiple benefit perceptions including functional utility, operational convenience, and hedonic satisfaction. Interestingly, Perceived Risk negatively influenced Attitude directly (*β* = −0.087, *p* = 0.016, supported) rather than through perceived value (*β* = −0.030, *p* = 0.264, not supported). This finding aligns with research on algorithm aversion demonstrating that professionals resist algorithmic recommendations even when algorithms outperform human judgment, with this reluctance rooted in concerns about losing control and professional autonomy (Berger et al., 2020).

Research on emotional responses to artificial intelligence reveals that employees’ affective reactions significantly shape attitudes, with negative emotions undermining positive attitude formation regardless of system functionality (Gkinko and Elbanna, 2022). These findings reinforce that risk perceptions constitute powerful barriers through direct emotional pathways rather than solely through cognitive value calculations, highlighting the importance of addressing psychological dimensions alongside functional considerations.

Contrary to expectations, neither Perceived Usefulness nor Perceived Ease of Use directly influenced Attitude. Instead, their effects operated entirely through perceived value, suggesting that in professional contexts, utilitarian and usability perceptions must first aggregate into comprehensive value assessments before shaping affective responses. The model explained substantial variance: 66.3% in perceived value, 83.8% in Attitude, and 74.0% in Intention to Use, demonstrating excellent explanatory power for the integrated framework.

H1 (PEOU → attitude) and H2 (PU → attitude) are unsupported. While TAM frequently predicts direct relationships between usefulness/ease and attitude, our findings imply that among Malaysian financial professionals, these beliefs might largely function by evaluating net benefits (perceived value) holistically before influencing attitude. Professionals may base their opinions in high-stakes decision-making situations more on whether AI is “worth it” overall given the accountability involved in their work, compliance requirements, and performance implications than on individual usability beliefs. This is in line with our findings that perceived value is strongly correlated with attitude, while PU, PEOU, and PE are significantly correlated with perceived value (all *p* < 0.001).

Unsupported H7 (perceived value → intention): The non-significant direct correlation between intention and perceived value suggests that attitude is the primary mechanism by which perceived value influences intention. This implies that unless cognitive value recognition is coupled by a positive affective evaluation (comfort, confidence, and psychological readiness to rely on AI), it may not be enough to create intention in the professional adoption of AI decision tools. The substantial correlation between attitude and intention (*β* = 0.773, *p* < 0.001) and the strong correlation between attitude and perceived value (*β* = 0.675, *p* < 0.001) all support this interpretation.

Unsupported H6 (perceived risk → perceived value): Perceived risk is not significantly correlated with perceived value, but it is negatively correlated with attitude (*β* = −0.087, *p* = 0.016). This suggests that risk may function more through affective reactions (anxiety, distrust, perceived accountability exposure) than through cognitive cost–benefit valuation. To put it another way, professionals may still see the benefits and overall worth of AI, but they may also be hesitant or uneasy because of the perceived risks. This might negatively impact attitude and indirectly impair intention.

### Theoretical implications

This study fills a specific gap in the literature on AI adoption: there is not much empirical data explaining how an integrated TAM–VAM framework functions among financial professionals in an emerging economy setting, especially when it comes to AI decision-support adoption, which explains AI adoption among professional employees in Malaysia’s financial sector. In addition to validating linkages, the results improve TAM–VAM integration by demonstrating that utilitarian views (usefulness, simplicity of use) are primarily linked to attitude through perceived value rather than immediately translating into attitude in this context. The importance of emotional evaluation in professional AI decision adoption is further highlighted by the fact that perceived value does not directly predict intention; rather, its link with intention is realized through attitude. Theoretically, Malaysia’s financial sector is significant because it includes both more traditional institutions and digitally advanced FinTech subsectors, resulting in differences in professional risk sensitivity, governance expectations, and AI exposure—conditions that make value-based mechanisms particularly important. While each model has been extensively validated independently, their integration provides more comprehensive understanding by simultaneously capturing utilitarian perceptions and value-based trade-off assessments (Liao et al., 2022).

The research extends artificial intelligence adoption theory to the financial services sector in an emerging market context, addressing gaps in understanding adoption dynamics beyond Western developed economies. By explaining 74 % of variance in adoption intentions, this study validates that these theoretical mechanisms possess cross-cultural applicability. This contribution is particularly timely given Malaysia’s ongoing digital transformation within its financial sector (Mohd Rashid et al., 2023). The research advances understanding of mediation mechanisms by revealing that perceived value and attitude serve as critical mediators translating antecedent perceptions into behavioral intentions, rather than exerting only direct effects. The research also contributes methodologically by demonstrating PLS-SEM’s effectiveness for testing complex mediation models in professional technology adoption contexts.

Much technology adoption research focuses on Western developed economies or consumer applications, not professional organizational contexts (Dwivedi et al., 2021a,b). The validation of multiple indirect effects via perceived value and attitude confirms the robustness of these analytical techniques for studying technology acceptance among working professionals (Klein et al., 2023).

### Practical implications

To guide AI implementation in financial institutions, prioritize fostering a positive Attitude, the strongest predictor of intention, via hands-on demos, pilot programs, and showcasing peer success. Training should cover both technical and psychological readiness. Since perceived value impacts attitude, clearly communicate AI’s value proposition: better decision accuracy, time savings, and competitive edge. Implementation should couple functional benefits with engaging, user-friendly interfaces. To counter risk perceptions that harm attitudes, ensure transparency in algorithmic processes, maintain human oversight, establish accountability, and provide robust data security.

Technology vendors developing artificial intelligence solutions for financial institutions should prioritize user-centered design emphasizing intuitive interfaces and straightforward functionality, as ease of use contributes significantly to perceived value. Products should deliver tangible usefulness improvements while incorporating engaging interaction elements. Vendors should provide comprehensive implementation support addressing both technical integration and psychological adoption barriers. For policymakers, findings suggest that regulatory frameworks supporting artificial intelligence adoption in Malaysia’s financial sector should balance innovation encouragement with appropriate safeguards addressing professionals’ risk concerns.

Bank Negara Malaysia and relevant authorities could develop guidelines clarifying accountability, data protection standards, and algorithmic transparency requirements to build confidence while fostering innovation. Recent developments in generative artificial intelligence, particularly large language models like ChatGPT, make these recommendations especially.

urgent. Research on organizational implications highlights challenges spanning ethical considerations, workforce transformation needs, and regulatory compliance (Dwivedi, 2023). Organizations need to foster collaborative human-AI partnerships, viewing algorithms as teammates that augment human judgment (Seeber et al., 2020). Malaysian financial institutions must implement strategies beyond technology acquisition, focusing on comprehensive organizational change management: cultural adaptation, role redefinition, skill development, and building trust between professionals and intelligent systems.

### Practical implications for financial institutions and technology

Financial institutions should invest in interventions that foster positive attitudes toward AI, such as supervised pilot programs, hands-on demonstrations, and role-based training that boosts confidence in utilizing AI in decision workflows, since attitude is the best predictor of intention (*β* = 0.773, *p* < 0.001). Institutions should convey value in ways that also promote psychological readiness because perceived value is influenced by attitude (e.g., presenting successful use cases, describing how AI supports—not replaces—professional judgement). Employees should be able to see governance mechanisms (data protection, audit trails, explicit accountability norms, human oversight) to lower fear and increase acceptance because perceived risk is inversely correlated with attitude. Vendors should give priority to workflow-fit design, intuitive interfaces, and explainability features in order to minimize cognitive effort and improve perceived value, as ease of use and usefulness are closely correlated with perceived value.

### Limitations

This study has several limitations. First, the research employed a convenience sampling approach to recruit participants from Malaysia’s financial industry. While this enabled practical data collection from professional employees meeting eligibility criteria, it limits generalizability to the broader Malaysian financial professional population. Future research employing probability sampling such as stratified random sampling would enhance representativeness and external validity. FinTech professionals were over-represented (50.5% of respondents) as a result of the sample strategy. Compared to experts in traditional banking and insurance, this segment is probably more exposed to AI and has more positive sentiments toward it. As a result, assessments of attitude and intention can be exaggerated in comparison to the larger Malaysian financial industry, limiting their external validity and generalizability. To increase representativeness, stratified sampling across subsectors should be used in future studies.

Second, this study intentionally excluded demographic variables such as age, gender, education level, and years of professional experience from data collection. While this decision focused attention on professional context variables and artificial intelligence-related perceptions consistent with the study’s theoretical focus on Technology Acceptance Model and Value-Based Adoption Model constructs, it limits ability to examine whether adoption mechanisms differ across demographic subgroups. Research suggests that age and gender may moderate technology adoption processes, with younger professionals potentially exhibiting different risk tolerance or ease-of-use expectations compared to older professionals, and gender potentially influencing risk or usefulness evaluations (Venkatesh et al., 2003). The absence of these demographic controls precludes investigation of such potential differences.

Third, the cross-sectional design captures perceptions at a single time point, preventing assessment of how adoption determinants evolve with actual usage experience. Fourth, the study measures behavioral intentions rather than actual usage behavior, introducing potential intention- behavior gaps. Fifth, geographic limitation to Malaysia may constrain transferability to other.

countries with different regulatory environments and cultural values. Sixth, exclusive focus on financial services limits insights about artificial intelligence adoption in other industries.

### Future research directions

Future research should employ longitudinal designs tracking professionals over time to understand how perceptions evolve with experience (Klein et al., 2023). Future research should investigate the automation-augmentation paradox—how AI simultaneously automates tasks and augments capabilities—to understand professional navigation of these tensions (Raisch and Krakowski, 2020). Comparative cross-country studies would test cultural generalizability, and extending research to other industries would test framework applicability.

Future research should explore the role of potential moderating variables that could influence the strength or direction of observed relationships. Specifically, individual difference factors—such as technical expertise, previous technology experience, or personality traits like openness to innovation—warrant investigation, as they may condition how strongly factors like perceived usefulness or perceived risk impact technology adoption decisions. Organizational factors including management support, training quality, or organizational culture could similarly moderate adoption pathways (Dwivedi et al., 2021a, 2021b). Specifically, future research should collect demographic variables including age, gender, education level, and years of professional experience to investigate whether adoption patterns differ across demographic subgroups. For instance, examining whether older versus younger professionals exhibit different weights for perceived risk versus perceived usefulness, or whether gender influences the importance of perceived ease of use versus perceived enjoyment, would provide insights for designing demographically-tailored implementation strategies. Such investigations would address the limitation of the present study which focused exclusively on professional context variables while excluding demographic characteristics. Qualitative research including interviews or focus groups could provide richer insights into lived experiences (Mohd Rashid et al., 2023). Finally, examining additional constructs such as trust in artificial intelligence, organizational support, or social influence might provide additional explanatory power. Research on AI in organizations identifies several other promising directions worth pursuing (Benbya et al., 2020).

## Conclusion

This research examined factors associated with professional employees’ intentions to adopt artificial intelligence in business decision-making within Malaysia’s financial industry through an integrated TAM-VAM framework. Analysis of data from 210 professionals revealed that the framework explains 74% of variance in adoption intentions, with attitude emerging as the strongest predictor and perceived value serving as a critical mediator. The findings provide theoretical contributions validating model integration in emerging market professional contexts and offer practical guidance for financial institutions, technology vendors, and policymakers. These findings should be interpreted as predictive associations given the cross-sectional design and the non-probability sampling approach.

While limitations including convenience sampling and cross-sectional design constrain generalizability, this research advances understanding of artificial intelligence adoption mechanisms in professional settings and provides empirical foundations for stakeholders seeking to enhance adoption effectiveness in Malaysia’s rapidly digitalizing financial sector.

## Data Availability

The original contributions presented in the study are included in the article/supplementary material, further inquiries can be directed to the corresponding authors.
